# A subanalysis of Japanese patients in a randomized, double-blind, placebo-controlled, phase 3 trial of nivolumab for patients with advanced gastric or gastro-esophageal junction cancer refractory to, or intolerant of, at least two previous chemotherapy regimens (ONO-4538-12, ATTRACTION-2)

**DOI:** 10.1007/s10120-018-0899-6

**Published:** 2018-12-01

**Authors:** Ken Kato, Taroh Satoh, Kei Muro, Takaki Yoshikawa, Takao Tamura, Yasuo Hamamoto, Keisho Chin, Keiko Minashi, Masahiro Tsuda, Kensei Yamaguchi, Nozomu Machida, Taito Esaki, Masahiro Goto, Yoshito Komatsu, Takako Eguchi Nakajima, Naotoshi Sugimoto, Kazuhiro Yoshida, Eiji Oki, Tomohiro Nishina, Akihito Tsuji, Hirofumi Fujii, Kenji Kunieda, Soh Saitoh, Yasushi Omuro, Mizutomo Azuma, Yasuo Iwamoto, Keisei Taku, Sachio Fushida, Li-Tzong Chen, Yoon-Koo Kang, Narikazu Boku

**Affiliations:** 1 Division of Gastrointestinal Medical Oncology, National Cancer Center Hospital, 5-1-1, Tsukiji, Chuo-ku, Tokyo, 104-0045 Japan; 2 Department of Frontier Science for Cancer and Chemotherapy, Osaka University Graduate School of Medicine, Suita, Japan; 3 Department of Clinical Oncology, Aichi Cancer Center Hospital, Nagoya, Japan; 4 Department of Gastrointestinal Surgery, Kanagawa Cancer Center, Yokohama, Japan; 5 Department of Medical Oncology, Kindai University, Faculty of Medicine, Osaka, Japan; 6 Keio Cancer Center, School of Medicine, Keio University, Tokyo, Japan; 7 Department of Gastroenterology, Cancer Institute Hospital of the Japanese Foundation for Cancer Research, Tokyo, Japan; 8 Clinical Trial Promotion Department, Chiba Cancer Center, Chiba, Japan; 9 Department of Gastroenterological Oncology, Hyogo Cancer Center, Akashi, Japan; 10 Department of Gastroenterology, Saitama Cancer Center, Saitama, Japan; 11 Division of Gastrointestinal Oncology, Shizuoka Cancer Center, Shizuoka, Japan; 12 Department of Gastrointestinal and Medical Oncology, National Hospital Organization Kyushu Cancer Center, Fukuoka, Japan; 13 Cancer Chemotherapy Center, Osaka Medical College Hospital, Takatsuki, Japan; 14 Division of Cancer Chemotherapy, Hokkaido University Hospital Cancer Center, Sapporo, Japan; 15 Department of Clinical Oncology, St. Marianna University School of Medicine, Kawasaki, Japan; 16 Department of Medical Oncology, Osaka International Cancer Institute, Osaka, Japan; 17 Department of Surgical Oncology, Gifu University Graduate School of Medicine, Gifu, Japan; 18 Department of Surgery and Science, Graduate School of Medical Sciences, Kyushu University, Fukuoka, Japan; 19 Department of Gastrointestinal Medical Oncology, National Hospital Organization Shikoku Cancer Center, Matsuyama, Japan; 20 Department of Medical Oncology, Kobe City Medical Center General Hospital, Kobe, Japan; 21 Department of Clinical Oncology, Cancer Center Jichi Medical University Hospital, Shimotsuke, Japan; 22 Department of Medical Oncology, Saku Central Hospital Advanced Care Center, Saku, Japan; 23 Internal Medicine, Misawa City Hospital, Misawa, Japan; 24 Department of Medical Oncology, Tokyo Metropolitan Cancer and Infectious Diseases Center Komagome Hospital, Tokyo, Japan; 25 Department of Gastroenterology, Kitasato University School of Medicine, Sagamihara, Japan; 26 Medical Oncology, Hiroshima City Hiroshima Citizens Hospital, Hiroshima, Japan; 27 Medical Oncology, Shizuoka General Hospital, Shizuoka, Japan; 28 Gastroenterological Surgery, Kanazawa University Hospital, Kanazawa, Japan; 29 National Institute of Cancer Research, National Health Research Institutes, Tainan, Taiwan, Republic of China; 30 Department of Internal Medicine, National Cheng Kung University Hospital, National Cheng Kung University, Tainan, Taiwan, Republic of China; 31 Department of Oncology, Asan Medical Center, University of Ulsan College of Medicine, Seoul, South Korea; 32 Present Address: Department of Gastric Surgery, National Cancer Center Hospital, Tokyo, Japan; 33 Present Address: Department of Medical Oncology, Kindai University Nara Hospital, Ikoma, Japan; 34 Present Address: Department of Gastroenterology, Cancer Institute Hospital of the Japanese Foundation for Cancer Research, Tokyo, Japan; 35 Present Address: Department of Medical Oncology, Kagawa University, Takamatsu, Japan

**Keywords:** Gastric cancer, Gastro-esophageal junction cancer, Japan, Nivolumab

## Abstract

**Background:**

Nivolumab, an anti-programmed death-1 agent, showed survival benefits in Asian patients, including Japanese, with gastric/gastro-esophageal junction (G/GEJ) cancer. We report the analysis of the Japanese subpopulation from ATTRACTION-2 that evaluated nivolumab versus placebo in unresectable advanced or recurrent G/GEJ cancer after ≥ 2 chemotherapy regimens.

**Methods:**

Data from the Japanese subpopulation in the randomized, double-blind, placebo-controlled, phase 3 trial were analyzed (data cutoff, February 25, 2017). Primary endpoint was overall survival (OS); secondary endpoints included progression-free survival (PFS) and objective response rate (ORR).

**Results:**

Among the overall study population of 493 patients, 226 (nivolumab 152; placebo 74) were enrolled from 28 sites in Japan. In the Japanese subset, median OS was longer with nivolumab versus placebo (5.4 months, 95% CI 4.6–7.4 versus 3.6 months, 95% CI 2.8–5.0). The risk of death was lower in the nivolumab versus placebo group (hazard ratio 0.58, 95% CI 0.42–0.78; *p* = 0.0002). Incidences of serious adverse events were 23% (35/152) and 25% (18/72) in the nivolumab and placebo groups, respectively. In the Japanese ITT population, 22% of nivolumab-treated and 28% of placebo-treated patients received prior ramucirumab treatment. Overall, clinical activity of nivolumab was observed regardless of prior ramucirumab use. In the nivolumab group, ORR and PFS were numerically higher in patients with prior ramucirumab use than in those without.

**Conclusions:**

In the Japanese subpopulation, patients receiving nivolumab had longer OS, similar to the overall population, with a manageable safety profile. The interaction between nivolumab and ramucirumab will be clarified in ongoing clinical trials.

**Electronic supplementary material:**

The online version of this article (10.1007/s10120-018-0899-6) contains supplementary material, which is available to authorized users.

## Introduction

In Japan, gastric cancer was the third leading cause of cancer-related death in 2016 and the most common malignancy in 2013 [1]. In general, treatment options include cytotoxic chemotherapy with addition of biologics for advanced gastric cancer. Since cross-over use of paclitaxel and irinotecan in second- and third-line chemotherapy was considered to contribute to a favorable overall survival (OS) in the WJOG 4007 study [2] compared with other studies outside Japan, irinotecan monotherapy is recommended as third-line therapy in the 2018 Gastric Cancer Treatment Guidelines by the Japanese Gastric Cancer Association (evidence level B) [3]. Regardless of these treatment options from first- to third-line treatment, the prognosis for advanced gastric cancer patients is poor, with a median OS of 13–14 months [4, 5]. Therefore, development of new therapies for patients with advanced gastric or gastro-esophageal junction (G/GEJ) cancer is warranted.

Inhibition of immune checkpoints is a proven therapeutic approach for many cancers. It includes the receptor–ligand system targeting programmed death-1 (PD-1), which is a cell surface receptor that blocks antitumor T-cell activity [6] after binding with programmed death-ligand 1 (PD-L1), which is expressed in 25–65% of gastric cancers and associated with tumor size, lymph node metastasis, and a shorter median survival [7–9]. Immuno-oncology agents, which block binding of PD-1 and PD-L1, are also being evaluated for G/GEJ cancer. The phase 1/2 CheckMate 032 trial reported clinical activity of nivolumab, alone or in combination with ipilimumab, an anti-cytotoxic T-lymphocyte–associated protein-4 antibody, in the gastric cohort with chemotherapy-refractory advanced G/GEJ/esophageal cancer [10, 11]. The phase 3 ATTRACTION-2 (ONO-4538-12) study conducted in Japan, Taiwan, and South Korea was also designed to investigate the efficacy and safety of nivolumab in heavily pretreated patients unselected for PD-L1 tumor expression. The results showed survival benefit with nivolumab versus placebo (median OS, 5.3 months vs 4.1 months; 12-month survival rates, 26.2% vs 10.9%, respectively), indicating that nivolumab can be a new treatment option for heavily pretreated patients with advanced G/GEJ cancer [12]. Therefore, nivolumab has been added as recommended third- or later-line therapy in the 2018 guideline (evidence level A) based on the prolonged OS in patients with advanced gastric cancer with failure of ≥ 2 lines of chemotherapy (ATTRACTION-2 study) [3, 12]. Further, the National Comprehensive Cancer Network guideline also recommends pembrolizumab as a third-line or subsequent therapy for recurrent, unresectable locally advanced, or metastatic gastric adenocarcinoma with PD-L1 expression [13].

However, the treatment strategy for advanced gastric cancer in Japan is somewhat different from that in Taiwan and South Korea. In Japan, the anti-vascular endothelial growth factor (VEGF) receptor 2 antibody ramucirumab, which is covered by National Health Insurance since 2015, has been widely used in combination with paclitaxel in second-line therapy. Thereafter, third-line chemotherapy is common if the patient’s condition is good. Several papers have reported that inhibition of VEGF signals changes the tumor microenvironment [14–18], which might have some influence on nivolumab efficacy. Therefore, we additionally analyzed data from the Japanese subpopulation to explore the impact of prior ramucirumab use on the efficacy of nivolumab.

## Methods

### Study design and patients

Data from Japanese patients enrolled in the randomized, double-blind, placebo-controlled, phase 3 ATTRACTION-2 trial (49 clinical sites in Japan, South Korea, and Taiwan) were analyzed. As a follow-up/update of ATTRACTION-2, the data cutoff date was February 25, 2017 for the Japanese subpopulation. The study design and results from the overall population have been previously reported [12].

### Procedures

Patients received nivolumab 3 mg/kg or placebo infusion every 2 weeks for each 6-week cycle (three infusions per 6-week cycle). Tumor assessments were performed after every 6-week treatment cycle for ten cycles (approximately 14 months). Thereafter, tumor assessments were performed after every two treatment cycles (12 weeks) until discontinuation of study treatment. A final assessment was also performed at the end-of-treatment examination. All tumor assessments were performed using computed tomography or magnetic resonance imaging according to the Response Evaluation Criteria in Solid Tumors guidelines version 1.1 [19]. The details have been described previously [12].

Adverse events (AEs) were assessed according to the National Cancer Institute Common Terminology Criteria for AEs version 4.0 [20] during the treatment period and for 28 days after end of treatment.

Tumor tissue collection was not compulsory; available tumor samples, which were collected at baseline, were evaluated retrospectively for PD-L1 tumor expression. Tumors with immunohistochemical staining in ≥ 1% of tumor cells, as assessed at a central laboratory (28-8 pharmDx assay; Dako, Carpinteria, CA, USA), were defined as positive.

### Outcomes

The primary endpoint was OS. Secondary efficacy endpoints included progression-free survival (PFS), objective response rate (ORR; the proportion of patients with confirmed complete response [CR] and partial response [PR]), disease control rate (DCR; the proportion of patients with confirmed CR, PR, and stable disease [SD]), duration of response (DOR), time to response, best overall response (CR, PR, SD, and progressive disease [PD]), and maximum percentage change from baseline in the sum of diameters of target lesions (% tumor shrinkage). PD-L1 expression status in patients with available tumor tissue sample was evaluated as an exploratory analysis. Safety endpoints included AEs and treatment-related AEs.

### Statistical analysis

Survival analyses were performed in the intention-to-treat (ITT) population, defined as all patients who were randomly assigned to the study treatment. Response rate was calculated in patients with measurable target lesions at baseline (response assessment population). Safety analyses were completed using data from all patients who received at least one dose of study treatment (safety population). The estimations of the OS and PFS rates were derived from the Kaplan–Meier estimates and the corresponding confidence intervals (CIs) were derived based on the Greenwood formula for variance and on log–log transformation. OS and PFS were compared using the stratified log-rank test with a one-sided significance level of 0.025. For best overall response, the exact 95% CI was calculated using the Clopper–Pearson method. Calculation of *p* value was conducted using the Cochran–Mantel–Haenszel test; SAS software (versions 9.3 and 9.4) was used for all statistical analyses. Other information has been reported previously [12].

## Results

### Patient disposition and baseline characteristics

Overall, 226 (nivolumab 152; placebo 74) of the 493 patients in ATTRACTION-2 were enrolled from 28 study sites in Japan. The safety population comprised 224 patients (nivolumab 152; placebo 72), and the response assessment population comprised 189 patients (nivolumab 129; placebo 60) (Supplementary Fig. 1). Baseline characteristics of the Japanese patients were well balanced between the treatment arms (Table 1). A total of 55 patients (nivolumab 34; placebo 21) were treated with ramucirumab prior to study entry.

**Table 1:** Baseline patient characteristics of subgroup of Japanese patients

	Nivolumab 3 mg/kg (*N* = 152)	Placebo (*N* = 74)
Male	111 (73)	57 (77)
Female	41 (27)	17 (23)
Age (years) median (min, max)	65 (20, 83)	66 (28, 79)
Patients aged < 65 years	68 (45)	28 (38)
Country		
Japan	152	74
Korea	–	–
Taiwan	–	–
Eastern Cooperative Oncology Group performance status		
0	64 (42)	31 (42)
1	88 (58)	43 (58)
Organs with metastases		
< 2	43 (28)	22 (30)
≥ 2	109 (72)	52 (70)
Site of metastases		
Lymph node	129 (85)	60 (81)
Peritoneum	28 (18)	15 (20)
Liver	35 (23)	17 (23)
Lung	11 (7)	4 (5)
Pleura	1 (1)	1 (1)
Adrenal glands	0	2 (3)
Bone	3 (2)	3 (4)
Other	8 (5)	7 (10)
Previous treatment regimens^a^		
2	11 (7)	3 (4)
3	57 (38)	26 (35)
≥ 4	84 (55)	45 (61)
Previous therapies		
Any	152 (100)	74 (100)
Pyrimidine analogs	152 (100)	74 (100)
Platinum	138 (91)	71 (96)
Taxanes	150 (99)	72 (97)
Irinotecan	137 (90)	70 (95)
Ramucirumab	34 (22)	21 (28)
Previous gastrectomy		
No	56 (37)	31 (42)
Yes	96 (63)	43 (58)

Data shown are *n* (%) unless otherwise stated

^a^Includes treatments received in the adjuvant setting

### Exposure and subsequent pharmacotherapy in the Japanese subpopulation

The median (range [min–max]) duration of treatment was 2.2 (0.0–24.4) months with nivolumab and 1.0 (0.0–26.3) months with placebo. Overall, the relative dose intensity of nivolumab was ≥ 90% to < 110% in 82.9% of patients. Details of study drug exposure and administration are presented in Supplementary Table 1. At data cutoff, study treatment was permanently discontinued in 143 patients in the nivolumab group and in 71 patients in the placebo group. Reasons for treatment discontinuation in the nivolumab versus placebo group were disease progression (114 [75%] vs 53 [73.6%]), worsening of clinical symptoms judged as PD (32 [21.1%] vs 17 [23.6%]), onset of grade ≥ 2 interstitial lung disease (4 [2.6%] vs 0 [0%]), physician’s discretion (6 [3.9%] vs 2 [2.8%]), treatment withheld for > 6 weeks due to AEs (2 [1.3%] vs 1 [1.4%]), and other reasons (4 [2.6%] vs 5 [6.9%]), respectively.

Following study treatment discontinuation, 53.3% (81/152) and 44.6% (33/74) of patients in the nivolumab and placebo groups, respectively, received subsequent anticancer treatment (pharmacotherapy, 30.9% [47/152] vs 24.3% [18/74]; surgery, 19.7% [30/152] vs 10.8% [8/74]; and radiotherapy, 9.2% [14/152] vs 13.5% [10/74], respectively; Supplementary Table 2).

### Efficacy in the Japanese subpopulation

#### Overall survival

**Fig. 1:**
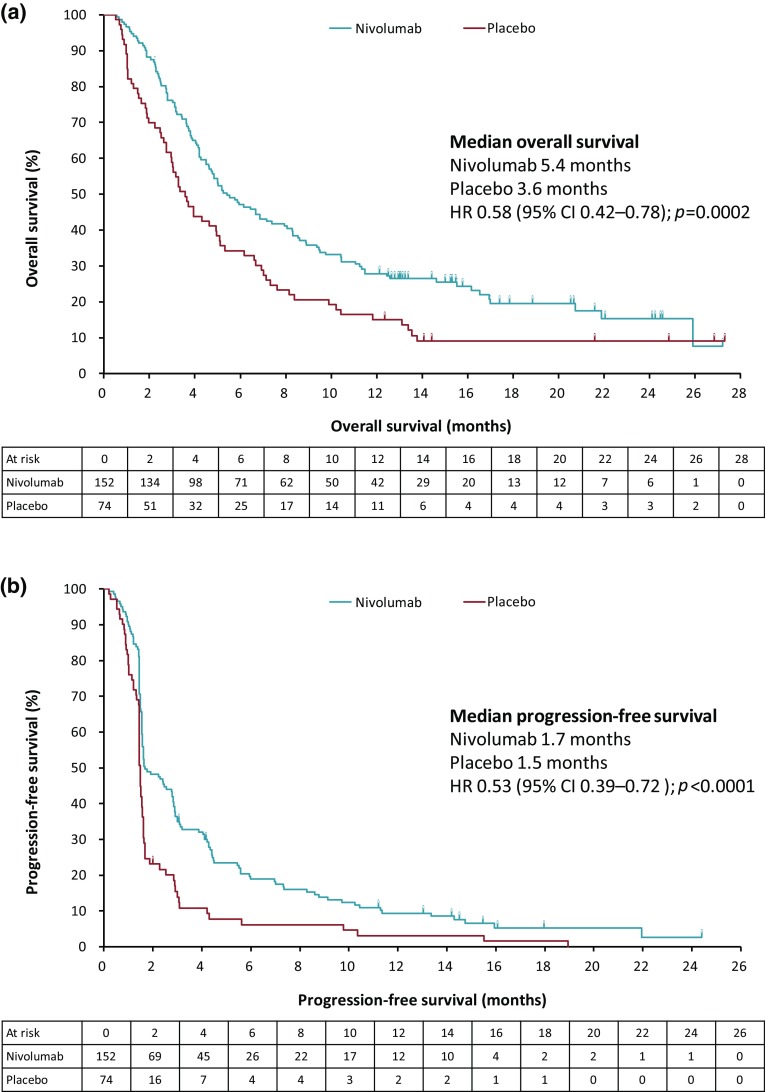
Kaplan–Meier plots of **a** overall survival and **b** progression-free survival (Japanese ITT population). *CI* Confidence interval, *HR* hazard ratio, *ITT* intention-to-treat

In the Japanese ITT population, as of February 25, 2017, 186 (82.3%) deaths had occurred (nivolumab 120 [78.9%]; placebo 66 [89.2%]). Median follow-up in the surviving patients was 16.6 months (interquartile range [IQR] 13.0–20.6; *n* = 32) in the nivolumab group and 17.7 months (IQR 13.2–25.8; *n* = 8) in the placebo group. By Kaplan–Meier analysis, the median OS was longer in the nivolumab versus placebo group (5.4 months, 95% CI 4.6–7.4 versus 3.6 months, 95% CI 2.8–5.0; Fig. 1a; Table 2). The risk of death was significantly lower in the nivolumab versus placebo group (hazard ratio [HR] 0.58, 95% CI 0.42–0.78; *p* = 0.0002). The OS rates were higher in the nivolumab versus placebo group at 6, 12, and 18 months (Table 2). Subgroup analyses of OS according to selected disease characteristics consistently favored nivolumab over placebo (Supplementary Fig. 2).

**Table 2:** Median overall survival and progression-free survival rates at 3, 6, 9, 12, 18, and 24 months (Japanese ITT population)

	Nivolumab 3 mg/kg (*N* = 152)	Placebo (*N* = 74)
OS rate, % (95% CI)		
At 3 months	76.2 (68.6–82.2)	58.9 (46.8–69.2)
At 6 months	47.1 (38.9–54.8)	34.2 (23.7–45.1)
At 9 months	35.8 (28.2–43.4)	20.5 (12.2–30.4)
At 12 months	27.8 (21.0–35.1)	15.1 (8.0–24.2)
At 18 months	19.5 (13.0–27.0)	9.0 (3.8–17.1)
PFS rate, % (95% CI)		
At 3 months	35.0 (27.3–42.8)	13.9 (6.9–23.2)
At 6 months	19.0 (13.0–25.9)	6.2 (2.0–13.7)
At 9 months	13.9 (8.8–20.2)	6.2 (2.0–13.7)
At 12 months	9.4 (5.2–15.0)	3.1 (0.6–9.5)
At 18 months	5.2 (2.1–10.5)	1.5 (0.1–7.3)

The estimation of the OS rate was derived from the Kaplan–Meier estimate and corresponding CI was derived based on Greenwood formula for variance and on log–log transformation

The estimation of the PFS rate was derived from the Kaplan–Meier estimate and corresponding CI was derived based on Greenwood formula for variance and on log–log transformation

1 month = 30.4375 days

*CI* Confidence interval, *ITT*, intention-to-treat, *OS* overall survival, *PFS* progression-free survival

#### Progression-free survival

By Kaplan–Meier analysis, PFS was significantly longer in the nivolumab versus placebo group at all follow-up time points (Fig. 1b; Table 2). The median PFS was 1.7 months (95% CI 1.6–2.8) in the nivolumab group versus 1.5 months (95% CI 1.5–1.6) in the placebo group. The 6-month PFS rate was higher in the nivolumab versus placebo group (19.0%, 95% CI 13.0–25.9 versus 6.2%, 95% CI 2.0–13.7). The risk of disease progression was lower in the nivolumab versus placebo group (HR 0.53, 95% CI 0.39–0.72; *p* < 0.0001).

#### Response

Patients with a confirmed response to nivolumab showed a median time to response of 1.7 months (min–max, 1.4–7.0). The median DOR was 14.5 months (95% CI 8.3–not available [NA]), and ORR was 14.0% (18/129) in patients treated with nivolumab. DCR was 45.0% (58/129 patients) in the nivolumab group versus 23.3% (14/60 patients) in the placebo group (odds ratio 2.87, 95% CI 1.40–5.88; Table 3 and Supplementary Fig. 3).

**Table 3:** Best overall response in Japanese subpopulation (response assessment population)

	Nivolumab (*N* = 129)	Placebo (*N* = 60)
Best overall response, *n* (%)		
CR	0	0
PR	18 (14.0)	0
SD	40 (31.0)	14 (23.3)
PD	61 (47.3)	40 (66.7)
NE	10 (7.8)	6 (10.0)
ORR		
ORR (CR + PR)	18 (14.0)	0
(95% CI)^a^	(8.5, 21.2)	(0.0, 6.0)
*p* value^b^	0.0023*	
DCR		
DCR (CR + PR + SD)	58 (45.0)	14 (23.3)
(95% CI)^a^	(36.2–54.0)	(13.4–36.0)
*p* value^b^	0.0037*	

Best overall response was determined solely by imaging assessment according to the RECIST Guideline Version 1.1

*CI* Confidence interval, *CR* complete response, *DCR* disease control rate, *ECOG* Eastern Cooperative Oncology group, *NE*, not evaluable, *ORR* objective response rate, *PD* progressive disease, *PR* partial response, *SD* stable disease

*p < 0.05

^a^Exact 95% CI was calculated using Clopper–Pearson method

^b^The calculation of *p* value was conducted using Cochran–Mantel–Haenszel test adjusted by the following three factors (interactive web response system): (1) location (Japan versus Korea versus Taiwan); (2) ECOG performance status score at baseline (0 versus 1); (3) number of organs with metastases (< 2 vs ≥ 2)

#### PD-L1 expression status

Baseline tumor samples were available for 61% (93/152) of patients in the nivolumab group and for 55% (41/74) of patients in the placebo group. PD-L1 expression was quantifiable in 91 and 41 patient samples in the nivolumab and placebo groups, respectively. Among them 13.2% (12/91) of patients in the nivolumab group and 19.5% (8/41) of patients in the placebo group had PD-L1–positive tumors. Although the patient numbers were low and the results did not reach significance, benefits of nivolumab (median OS, 6.14 months) were observed even in patients without PD-L1-positive tumors (HR 0.76, 95% CI 0.49–1.18) as compared to the median OS reported in this study.

### Safety in the Japanese subpopulation

Overall, AEs were reported more frequently in the nivolumab versus placebo group (Supplementary Table 3). All-cause AEs of any grade occurred in 84.9% (129/152) of patients in the nivolumab group versus 73.6% (53/72) in the placebo group; incidences of serious AEs were 23% (35/152) versus 25% (18/72), respectively.

Treatment-related AEs of any grade were reported in 56.6% (86/152) of patients in the nivolumab group versus 30.6% (22/72) in the placebo group; grade 3/4 treatment-related AEs were 15.8% (24/152) versus 9.7% (7/72), respectively. Incidences of serious treatment-related AEs were 13.2% (20/152) versus 9.7% (7/72), respectively.

The most commonly (> 5% incidence) reported all-grade treatment-related AEs were pruritus, diarrhea, rash, fatigue, nausea, malaise, and decreased appetite. The most commonly reported grade 3/4 AEs were decreased appetite and diarrhea in the nivolumab group and decreased appetite and fatigue in the placebo group (Table 4). The incidence of most frequent treatment-related serious AEs was low (≤ 2%) in the nivolumab group and included interstitial lung disease, type 1 diabetes mellitus, and colitis (Supplementary Table 4). Two deaths (1.3%; cardiac arrest, unknown cause) in the nivolumab group versus 1 death (1.4%; gastrointestinal perforation) in the placebo group were considered treatment-related AEs in the Japanese subpopulation. Treatment was discontinued due to treatment-related AEs in 4.6% (7/152) versus 5.6% (4/72) of patients in the nivolumab versus placebo group, respectively.

**Table 4:** Incidence of treatment-related adverse events occurring in ≥ 5% of Japanese patients and additional treatment-related adverse events of special interest (safety population)

Adverse event, *n* (%)	Nivolumab (*N* = 152)		Placebo (*N* = 72)	
	Any grade	Grade 3/4	Any grade	Grade 3/4
All	86 (56.6)	24 (15.8)	22 (30.6)	7 (9.7)
Pruritus	17 (11.2)	0	1 (1.4)	0
Diarrhea	14 (9.2)	2 (1.3)	2 (2.8)	0
Malaise	12 (7.9)	0	6 (8.3)	0
Fatigue	11 (7.2)	1 (0.7)	4 (5.6)	2 (2.8)
Decreased appetite	10 (6.6)	3 (2.0)	4 (5.6)	1 (1.4)
Rash	10 (6.6)	0	2 (2.8)	0
Nausea	10 (6.6)	0	1 (1.4)	0
Additional treatment-related adverse events of special interest				
Interstitial lung disease	6 (3.9)	1 (0.7)	0	0
Rash maculo-papular	5 (3.3)	0	0	0
Colitis	2 (1.3)	1 (0.7)	0	0
Hypopituitarism	1 (0.7)	1 (0.7)	0	0
Pneumonitis	1 (0.7)	1 (0.7)	0	0
Hyperthyroidism	1 (0.7)	0	0	0
Thyroid disorder	1 (0.7)	0	0	0
Hepatic function abnormal	0	0	2 (2.8)	1 (1.4)
Acute hepatic failure	0	0	1 (1.4)	1 (1.4)
Acute hepatitis	0	0	0	0
Autoimmune thyroiditis	0	0	0	0

### Efficacy analysis of patients with prior ramucirumab treatment

**Fig. 2:**
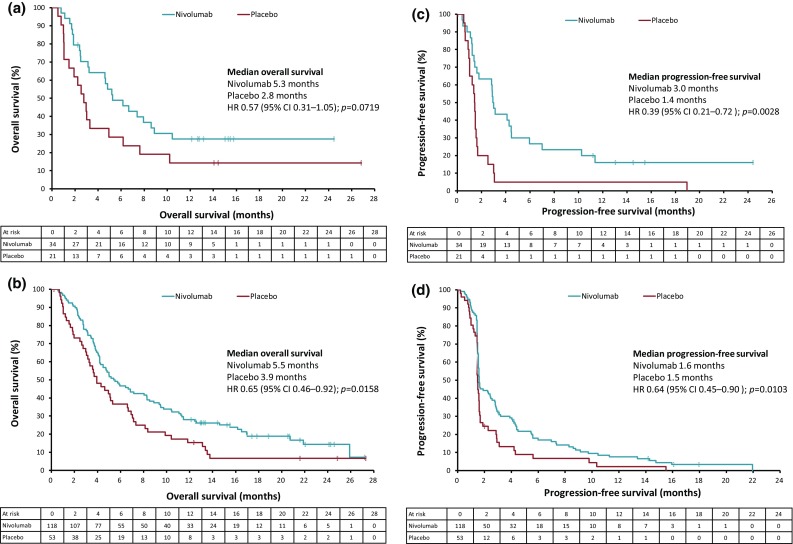
Kaplan–Meier plots of overall survival in **a** patients with prior ramucirumab treatment; **b** patients without prior ramucirumab treatment; and progression-free survival in **c** patients with prior ramucirumab treatment; **d** patients without prior ramucirumab treatment (ITT population). *CI* Confidence interval, *HR* hazard ratio, *ITT* intention-to-treat

A total of 34 and 21 patients received prior ramucirumab treatment in the nivolumab and placebo groups, respectively. The baseline characteristics were similar in patients with and without prior ramucirumab use, except for the variability observed in the number of prior regimens (Supplementary Table 5). The median OS was longer in the nivolumab versus placebo group in patients with (5.3 versus 2.8 months [HR 0.57, 95% CI 0.31–1.05]) and without (5.5 versus 3.9 months [HR 0.65, 95% CI 0.46–0.92]) prior ramucirumab treatment (Fig. 2a, b). The median PFS was longer in the nivolumab versus placebo group in patients with (3.0 versus 1.4 months [HR 0.39, 95% CI 0.21–0.72]) and without (1.6 versus 1.5 months [HR 0.64, 95% CI 0.45–0.90]) prior ramucirumab treatment (Fig. 2c, d). Among patients evaluable for tumor response, the ORR in the nivolumab group was higher in patients with (22.2% [6/27]) versus without (11.8% [12/102]) prior ramucirumab treatment. DCR was higher in the nivolumab versus placebo group in patients with (55.6% [15/27] vs 21.1% [4/19]) and without (42.2% [43/102] vs 24.4% [10/41]) previous ramucirumab treatment (Table 5). Furthermore, better clinical activity was observed in 25 of the 34 patients who received nivolumab directly after ramucirumab treatment. In the ITT (*n* = 25) and response assessment (*n* = 20) populations, for patients who received nivolumab directly after ramucirumab, the median OS, median PFS, ORR, and DCR were 6.7 months, 4.2 months, 25.0% (5/20), and 70% (14/20), respectively.

**Table 5:** Best overall response in Japanese subpopulation based on prior treatment with ramucirumab (response assessment population)

	With prior ramucirumab treatment		Without prior ramucirumab treatment	
	Nivolumab (*N* = 27)	Placebo (*N* = 19)	Nivolumab (*N* = 102)	Placebo (*N* = 41)^a^
Best overall response, *n* (%)				
CR	0	0	0	0
PR	6 (22.2)	0	12 (11.8)	0
SD	9 (33.3)	4 (21.1)	31 (30.4)	10 (24.4)
PD	7 (25.9)	13 (68.4)	54 (52.9)	27 (65.9)
NE	5 (18.5)	2 (10.5)	5 (4.9)	3 (7.3)
ORR				
ORR (CR + PR)	6 (22.2)	0	12 (11.8)	0
DCR				
DCR (CR + PR + SD)	15 (55.6)	4 (21.1)	43 (42.2)	10 (24.4)

Best overall response was determined solely by imaging assessment according to the RECIST Guideline Version 1.1

*CR* Complete response, *DCR* disease control rate, *NE* not evaluable, *ORR* objective response rate, *PD* progressive disease, *PR* partial response, *SD* stable disease

^a^One patient dropped out before placebo administration

## Discussion

We report the analysis of data from the Japanese subpopulation in the randomized, double-blind, placebo-controlled, phase 3 ATTRACTION-2 trial. The results of this subanalysis showed consistency in the efficacy of nivolumab between the Japanese subpopulation and the overall study population reported previously [12]. Of note, more Japanese patients had received ≥ 4 previous anticancer treatment regimens and their Eastern Cooperative Oncology Group (ECOG) performance status was better than that of the overall study population. In terms of efficacy, the OS, PFS, DOR, SD, and DCR were similar between the Japanese subpopulation and the overall study population, in both the nivolumab and placebo groups. The risks of death and disease progression were lower in the nivolumab group than in the placebo group and this was consistent throughout the follow-up period in both populations. Subgroup analyses of OS according to selected disease characteristics were also similar in both populations and consistently favored nivolumab over placebo (Supplementary Fig. 2).

A greater median OS has previously been reported with the biological agent ramucirumab as second-line treatment in Japanese/East Asian patients compared with Western patients with gastric cancer [21]. Furthermore, OS benefits with new agents over control treatments observed in non-Japanese/non-Asian populations were not confirmed in the Japanese/Asian subpopulation [21, 22]. It is considered that the high rate of post-discontinuation therapy (second-line or later) in the Japanese subpopulation does not allow differentiation of efficacy with new agents in these trials. Considering this difference in treatment strategy and clinical course, the significant improvement in OS and PFS with nivolumab, compared with the placebo group, in the Japanese subpopulation and East Asian patients from ATTRACTION-2 was a noteworthy outcome. Of note, although the Japanese subpopulation of ATTRACTION-2 received treatment in a late-line setting, the favorable outcome with nivolumab may be partly attributed to the better performance status of these patients, with 42% having an ECOG performance status of 0.

We observed that almost all patients who had received ramucirumab prior to nivolumab in ATTRACTION-2 were Japanese (55/57 patients). Therefore, an additional analysis exploring the impact of prior ramucirumab treatment on the efficacy of nivolumab treatment was performed in the Japanese subpopulation. In this analysis, nivolumab showed efficacy in both groups of patients, regardless of prior ramucirumab treatment, compared with placebo. The risks of death and disease progression were numerically better in patients with versus those without prior ramucirumab treatment. This could be attributed to VEGF signaling that is known to modify the tumor immunological environment with T-cell activation and Treg suppression [14–18]. Some reports have also shown positive clinical activity with immune-checkpoint inhibitors and antiangiogenic drugs in lung and renal cancer [23, 24]. This is supported by the observation that 25 of the 34 patients who received nivolumab directly after ramucirumab treatment reported better clinical efficacy. Therefore, it is speculated that ramucirumab might enhance the efficacy of nivolumab. However, this additional analysis by prior ramucirumab treatment was conducted in a small number of patients. Several clinical trials of the combination with ramucirumab and an anti-PD-1/PD-L1 antibody are underway, and will help validate our observations.

The median DOR with nivolumab in the Japanese population was 14.5 months (95% CI 8.3–NA). On the other hand, the DOR with nivolumab in the overall population was reported as 9.5 months (95% CI 6.14–9.82) [12]. Although the cutoff date was different, the DOR with nivolumab in the Japanese population appears to be longer than that of the overall population. The underlying reason for this is not clear; however, it may be partly attributed to the better performance status in the nivolumab-treated Japanese subpopulation (42% with a performance status of 0) compared with the nivolumab-treated overall population (29% with a performance status of 0) reported previously [12].

The safety profile of the Japanese subpopulation was similar to that of the overall study population [12], and no treatment-related AEs specific to the Japanese were observed.

The proportion of PD-L1–positive patients observed in our study was lower than that in the KEYNOTE-059 trial (PD-L1 positivity of 57%), which employed a combined positive score (including tumor cells, macrophages, and lymphocytes) [25]. This difference in proportion between KEYNOTE-059 and the current study could be due to the difference in the scoring method employed [26]. Moreover, the relationship between the PD-L1 expression score and response to therapy remains to be elucidated. We would also like to acknowledge that treatment efficacy could not be evaluated based on PD-L1 expression due to the limited number of patients in this subgroup analysis. Other biomarkers, including microsatellite instability or Epstein-Barr virus positivity, were not available in this analysis, which may be a limitation of this report.

Overall, there were no notable differences in the efficacy and safety outcomes with nivolumab between the Japanese and the overall populations, and no treatment-related AEs specific to the Japanese were observed.

## Conclusion

In the Japanese subpopulation, patients with advanced G/GEJ cancer treated with nivolumab had a manageable safety profile and longer OS, with early and durable responses, versus patients treated with placebo. Additionally, the benefit of sequential use of ramucirumab followed by nivolumab was observed in an exploratory analysis requiring further validation.

## Electronic supplementary material

Below is the link to the electronic supplementary material.

Supplementary material 1 (DOCX 88 KB)

## References

[1] Cancer Information Service, National Cancer Center, Japan. Cancer registry and statistics (article in Japanese). 2018. https://ganjoho.jp/reg_stat/statistics/stat/summary.html. Accessed 16 Apr 2018.

[2] Hironaka S, Ueda S, Yasui H, Nishina T, Tsuda M, Tsumura T (2013). Randomized, open-label, phase III study comparing irinotecan with paclitaxel in patients with advanced gastric cancer without severe peritoneal metastasis after failure of prior combination chemotherapy using fluoropyrimidine plus platinum: WJOG 4007 trial. J Clin Oncol.

[3] Japan Society of Gastric Cancer. Japanese gastric cancer treatment guidelines 2018 (article in Japanese). 2018. http://www.kanehara-shuppan.co.jp/books/detail.html?isbn=9784307203814. Accessed 16 Apr 2018.

[4] Yamada Y, Higuchi K, Nishikawa K, Gotoh M, Fuse N, Sugimoto N (2015). Phase III study comparing oxaliplatin plus S-1 with cisplatin plus S-1 in chemotherapy-naïve patients with advanced gastric cancer. Ann Oncol.

[5] Koizumi W, Narahara H, Hara T, Takagane A, Akiya T, Takagi M (2008). S-1 plus cisplatin versus S-1 alone for first-line treatment of advanced gastric cancer (SPIRITS trial): a phase III trial. Lancet Oncol.

[6] Sharma P, Allison JP (2015). Immune checkpoint targeting in cancer therapy: toward combination strategies with curative potential. Cell.

[7] Yuan J, Zhang J, Zhu Y, Li N, Tian T, Li Y (2016). Programmed death-ligand-1 expression in advanced gastric cancer detected with RNA in situ hybridization and its clinical significance. Oncotarget.

[8] Kawazoe A, Kuwata T, Kuboki Y, Shitara K, Nagatsuma AK, Aizawa M (2017). Clinicopathological features of programmed death ligand 1 expression with tumor-infiltrating lymphocyte, mismatch repair, and Epstein–Barr virus status in a large cohort of gastric cancer patients. Gastric Cancer.

[9] Zhang M, Dong Y, Liu H, Wang Y, Zhao S, Xuan Q (2016). The clinicopathological and prognostic significance of PD-L1 expression in gastric cancer: a meta-analysis of 10 studies with 1,901 patients. Sci Rep.

[10] Janjigian YY, Bendell JC, Calvo E, Kim JW, Ascierto PA, Sharma P (2016). CheckMate-032: Phase I/II, open-label study of safety and activity of nivolumab (nivo) alone or with ipilimumab (ipi) in advanced and metastatic (A/M) gastric cancer (GC). J Clin Oncol.

[11] Janjigian YY, Ott PA, Calvo E, Kim JW, Ascierto PA, Sharma P (2017). Nivolumab ± ipilimumab in pts with advanced (adv)/metastatic chemotherapy-refractory (CTx-R) gastric (G), esophageal (E), or gastroesophageal junction (GEJ) cancer: CheckMate 032 study. J Clin Oncol.

[12] Kang YK, Boku N, Satoh T, Ryu MH, Chao Y, Kato K (2017). Nivolumab in patients with advanced gastric or gastro-oesophageal junction cancer refractory to, or intolerant of, at least two previous chemotherapy regimens (ONO-4538-12, ATTRACTION-2): a randomised, double-blind, placebo-controlled, phase 3 trial. Lancet.

[13] National Comprehensive Cancer Network. NCCN Guidelines for Gastric Cancer V.5.2017. 2017. https://www.nccn.org/Common/FileManager.ashx?fileManagerId=96a310fb-6b85-4233-8817-0be95e19fc6f. Accessed 16 Apr 2018.

[14] Motz GT, Coukos G (2011). The parallel lives of angiogenesis and immunosuppression: cancer and other tales. Nat Rev Immunol.

[15] Della Porta M, Danova M, Rigolin GM, Brugnatelli S, Rovati B, Tronconi C (2005). Dendritic cells and vascular endothelial growth factor in colorectal cancer: correlations with clinicobiological findings. Oncology.

[16] Takahashi A, Kono K, Ichihara F, Sugai H, Fujii H, Matsumoto Y (2004). Vascular endothelial growth factor inhibits maturation of dendritic cells induced by lipopolysaccharide, but not by proinflammatory cytokines. Cancer Immunol Immunother.

[17] Liu XD, Hoang A, Zhou L, Kalra S, Yetil A, Sun M (2015). Resistance to antiangiogenic therapy is associated with an immunosuppressive tumor microenvironment in metastatic renal cell carcinoma. Cancer Immunol Res.

[18] Wallin JJ, Bendell JC, Funke R, Sznol M, Korski K, Jones S (2016). Atezolizumab in combination with bevacizumab enhances antigen-specific T-cell migration in metastatic renal cell carcinoma. Nat Commun.

[19] Eisenhauer EA, Therasse P, Bogaerts J, Schwartz LH, Sargent D, Ford R (2009). New response evaluation criteria in solid tumours: revised RECIST guideline (version 1.1). Eur J Cancer.

[20] National Cancer Institute. Common Terminology Criteria for Adverse Events (CTCAE) version 4.0. 2010. https://evs.nci.nih.gov/ftp1/CTCAE/CTCAE_4.03/Archive/CTCAE_4.0_2009-05-29_QuickReference_8.5x11.pdf. Accessed 16 Apr 2018.

[21] Shitara K, Muro K, Shimada Y, Hironaka S, Sugimoto N, Komatsu Y (2016). Subgroup analyses of the safety and efficacy of ramucirumab in Japanese and Western patients in RAINBOW: a randomized clinical trial in second-line treatment of gastric cancer. Gastr Cancer.

[22] Ohtsu A, Shah MA, Van Cutsem E, Rha SY, Sawaki A, Park SR (2011). Bevacizumab in combination with chemotherapy as first-line therapy in advanced gastric cancer: a randomized, double-blind, placebo-controlled phase III study. J Clin Oncol.

[23] Socinski MA, Jotte RM, Cappuzzo F, Orlandi F, Stroyakovskiy D, Nogami N (2018). Atezolizumab for first-line treatment of metastatic nonsquamous NSCLC. N Engl J Med.

[24] McDermott DF, Huseni MA, Atkins MB, Motzer RJ, Rini BI, Escudier B (2018). Clinical activity and molecular correlates of response to atezolizumab alone or in combination with bevacizumab versus sunitinib in renal cell carcinoma. Nat Med.

[25] Fuchs CS, Doi T, Jang RW, Muro K, Satoh T, Machado M (2018). Safety and efficacy of pembrolizumab monotherapy in patients with previously treated advanced gastric and gastroesophageal junction cancer: phase 2 clinical KEYNOTE-059 trial. JAMA Oncol.

[26] Kulangara K, Zhang N, Corigliano E, Guerrero L, Waldroup S, Jaiswal D (2018). Clinical utility of the combined positive score for programmed death ligand-1 expression and the approval of pembrolizumab for treatment of gastric cancer. Arch Pathol Lab Med.

