# Supplementary material for: A subanalysis of Japanese patients in a randomized, double-blind, placebo-controlled, phase 3 trial of nivolumab for patients with advanced gastric or gastro-esophageal junction cancer refractory to, or intolerant of, at least two previous chemotherapy regimens (ONO-4538-12, ATTRACTION-2)
Source: Gastric Cancer. 2018 Dec 1;22(2):344–54. doi: 10.1007/s10120-018-0899-6 (PMC6394726; doi:10.1007/s10120-018-0899-6)
Supplement: Supplementary file 1 — Supplementary material 1 (DOCX 88 KB) [file 10120_2018_899_MOESM1_ESM.docx]

**Supplementary material**

**Supplementary Fig. 1** Disposition of Japanese subpopulation

*ITT* intention-to-treat

^a^Multiple reasons for study treatment discontinuation could be provided. ^b^Unless study treatment withheld for more than 6 weeks for steroid tapering.

**Supplementary Fig. 2** Forest plot of overall survival according to patient subgroups (ITT population)

*CI* confidence interval, *ECOG* Eastern Cooperative Oncology Group, *ITT* intention-to-treat, *TNM* tumor node metastasis

**Supplementary Fig. 3** Waterfall plot of percentage tumor shrinkage in **a** nivolumab group and **b** placebo group (response assessment population)

**Supplementary Table 1** Extent of exposure and administration of nivolumab and placebo (safety population)

| **Analysis item** | **Japanese subpopulation** | | |
| --- | --- | --- | --- |
|  | **Nivolumab** | | **Placebo** |
|  | **(*N* = 152)** | | **(*N* = 72)** |
| Number of doses received |  | |  |
| Mean (SD) | 9.3 (10.6) | | 5.6 (9.2) |
| Median | 5.0 | | 3.0 |
| Min, max | 1, 52 | | 1, 53 |
| Duration of treatment (months)^a^ |  | |  |
| >6 | 32 (21.1) | | 5 (6.9) |
| >12 | 19 (12.5) | | 3 (4.2) |
| Mean (SD) | 4.10 (5.13) | | 2.28 (4.46) |
| Median | 2.20 | | 0.95 |
| Min, max | 0.0, 24.4 | | 0.0, 26.3 |
| Number of cycles^b^ |  | |  |
| 1 | 59 (38.8) | | 42 (58.3) |
| 2–3 | 52 (34.2) | | 25 (34.7) |
| 4–6 | 17 (11.2) | | 1 (1.4) |
| ≥7 | 24 (15.8) | | 4 (5.6) |
| Mean (SD) | 3.4 (3.6) | | 2.1 (3.1) |
| Median | 2.0 | | 1.0 |
| Min, max | 1, 18 | | 1, 19 |
| Cumulative dose (mg/kg)^c^ |  |  | |
| Mean (SD) | 27.96 (31.62) |  | |
| Median | 15.14 |  | |
| Min, max | 3.0, 158.0 |  | |
| Relative dose intensity (%)^d^ |  |  | |
| <50 | 0 |  | |
| 50–<70 | 3 (2.0) |  | |
| 70–<90 | 21 (13.8) |  | |
| 90–<110 | 126 (82.9) |  | |
| ≥110 | 2 (1.3) |  | |
| Mean (SD) | 95.98 (7.85) |  | |
| Median | 97.08 |  | |
| Min, max | 60.7, 112.6 |  | |

Data shown are *n* (%) unless otherwise stated.

^a^Duration of treatment (months) = (“date of the last dose” – “date of the first dose” +1)/30.4375

^b^The number of cycles was calculated for the cycle proceeding to the next cycle. The discontinued cycle or the cycle receiving no investigational product was also included.

^c^Cumulative dose of nivolumab (mg/kg) is sum of the actual doses (mg/kg) administered to a subject during the treatment period. Actual dose (mg/kg) at each time point was calculated by the following equation: actual dose (mg/kg) at each time point = “actual dose amount (mg)” / “recent weight (kg)”

^d^Relative dose intensity (%) = “cumulative dose (mg/kg)” / “[date of the last dose – date of the first dose + 14] (days) × 3 (mg/kg) / 14 (days)” × 100

**Supplementary Table 2** Post-progression anticancer therapies (Japanese subpopulation; ITT population).

|  | **Japanese subpopulation** | |
| --- | --- | --- |
| **Therapies, *n* (%)** | **Nivolumab** | **Placebo** |
|  | **(*N* = 152)** | **(*N* = 74)** |
| Any post-progression therapy | 81 (53.3) | 33 (44.6) |
| Radiotherapy | 14 (9.2) | 10 (13.5) |
| Surgery | 30 (19.7) | 8 (10.8) |
| Chemotherapy | 47 (30.9) | 18 (24.3) |
| Post-progression pharmacotherapy |  |  |
| Fluoropyrimidine | 17 (11.2) | 10 (13.5) |
| Platinum compound | 15 (9.9) | 9 (12.2) |
| Taxane | 14 (9.2) | 4 (5.4) |
| Irinotecan | 1 (0.7) | 1 (1.4) |
| Ramucirumab | 34 (22.4) | 12 (16.2) |
| Immunotherapy^a^ | 0 | 1 (1.4) |
| Other targeted therapies^b^ | 1 (0.7) | 1 (1.4) |

^a^Immunotherapy (not specified).

^b^Other targeted therapies (GDC-0068, JNJ42756493).

*ITT* intention-to-treat

**Supplementary Table 3** Incidence of all adverse events occurring in ≥5% of patients in the safety analysis population

| ***n* (%)** | **Japanese population** | | | |
| --- | --- | --- | --- | --- |
|  | **Nivolumab**  **(*N* = 152)** | | **Placebo**  **(*N*= 72)** | |
|  | **Any grade** | **Grade 3/4** | **Any grade** | **Grade 3/4** |
| Diarrhea | 30 (19.7) | 3 (2.0) | 5 (6.9) | 0 |
| Pruritus | 24 (15.8) | 0 | 4 (5.6) | 0 |
| Nausea | 23 (15.1) | 0 | 5 (6.9) | 1 (1.4) |
| Constipation | 22 (14.5) | 1 (0.7) | 2 (2.8) | 0 |
| Vomiting | 17 (11.2) | 1 (0.7) | 6 (8.3) | 0 |
| Decreased appetite | 16 (10.5) | 4 (2.6) | 10 (13.9) | 4 (5.6) |
| Pyrexia | 14 (9.2) | 0 | 8 (11.1) | 1 (1.4) |
| Malaise | 14 (9.2) | 0 | 8 (11.1) | 0 |
| Rash | 13 (8.6) | 0 | 4 (5.6) | 0 |
| Fatigue | 12 (7.9) | 1 (0.7) | 8 (11.1) | 3 (4.2) |
| Viral upper respiratory tract infection | 12 (7.9) | 0 | 4 (5.6) | 0 |
| Blood alkaline phosphatase increased | 8 (5.3) | 4 (2.6) | 0 | 0 |
| Hypothyroidism | 8 (5.3) | 0 | 1 (1.4) | 0 |
| Stomatitis | 8 (5.3) | 0 | 1 (1.4) | 0 |
| Weight decreased | 7 (4.6) | 1 (0.7) | 5 (6.9) | 0 |
| Aspartate aminotransferase increased | 7 (4.6) | 1 (0.7) | 1 (1.4) | 0 |
| Insomnia | 7 (4.6) | 0 | 5 (6.9) | 0 |
| Edema peripheral | 7 (4.6) | 0 | 0 | 0 |
| Gamma-glutamyltransferase increased | 6 (3.9) | 2 (1.3) | 0 | 0 |
| Anemia | 5 (3.3) | 4 (2.6) | 1 (1.4) | 0 |
| Alanine aminotransferase increased | 5 (3.3) | 1 (0.7) | 0 | 0 |
| Back pain | 5 (3.3) | 0 | 0 | 0 |
| Abdominal pain | 4 (2.6) | 0 | 2 (2.8) | 0 |
| Hypoalbuminemia | 3 (2.0) | 3 (2.0) | 1 (1.4) | 0 |
| Cough | 3 (2.0) | 0 | 2 (2.8) | 0 |
| Dyspepsia | 2 (1.3) | 0 | 0 | 0 |
| Hepatic function abnormal | 1 (0.7) | 1 (0.7) | 4 (5.6) | 3 (4.2) |
| Dyspnea | 1 (0.7) | 1 (0.7) | 0 | 0 |
| Ascites | 1 (0.7) | 1 (0.7) | 0 | 0 |
| Cystitis | 1 (0.7) | 0 | 4 (5.6) | 0 |
| Abdominal distension | 1 (0.7) | 0 | 0 | 0 |
| Asthenia | 0 | 0 | 0 | 0 |
| Malignant neoplasm progression | 0 | 0 | 0 | 0 |
| Blood bilirubin increased | 0 | 0 | 0 | 0 |
| Pleural effusion | 0 | 0 | 0 | 0 |

**Supplementary Table 4** Incidence of treatment-related serious adverse events in the safety analysis population

| ***n* (%)** | **Japanese population** | | | |
| --- | --- | --- | --- | --- |
|  | **Nivolumab**  **(*N* = 152)** | | **Placebo**  **(*N*= 72)** | |
|  | **Any grade** | **Grade 3/4** | **Any grade** | **Grade 3/4** |
| Interstitial lung disease | 3 (2.0) | 1 (0.7) | 0 | 0 |
| Type 1 diabetes mellitus | 2 (1.3) | 2 (1.3) | 0 | 0 |
| Colitis | 2 (1.3) | 1 (0.7) | 0 | 0 |
| Pneumonia | 1 (0.7) | 1 (0.7) | 1 (1.4) | 1 (1.4) |
| Upper gastrointestinal hemorrhage | 1 (0.7) | 1 (0.7) | 1 (1.4) | 1 (1.4) |
| Fatigue | 1 (0.7) | 1 (0.7) | 1 (1.4) | 1 (1.4) |
| Hypopituitarism | 1 (0.7) | 1 (0.7) | 0 | 0 |
| Dry eye | 1 (0.7) | 1 (0.7) | 0 | 0 |
| Dry mouth | 1 (0.7) | 1 (0.7) | 0 | 0 |
| Vomiting | 1 (0.7) | 1 (0.7) | 0 | 0 |
| Splenic infection | 1 (0.7) | 1 (0.7) | 0 | 0 |
| Pneumonitis | 1 (0.7) | 1 (0.7) | 0 | 0 |
| Pneumomediastinum | 1 (0.7) | 1 (0.7) | 0 | 0 |
| Decreased appetite | 1 (0.7) | 1 (0.7) | 0 | 0 |
| Sjogren’s syndrome | 1 (0.7) | 1 (0.7) | 0 | 0 |
| Dyspnea | 1 (0.7) | 1 (0.7) | 0 | 0 |
| Death | 1 (0.7) | 0 | 1 (0.7) | 0 |
| Urinary tract infection | 1 (0.7) | 0 | 0 | 0 |
| Cardiac arrest | 1 (0.7) | 0 | 0 | 0 |
| Atypical mycobacterial pneumonia | 1 (0.7) | 0 | 0 | 0 |
| Pneumothorax | 1 (0.7) | 0 | 0 | 0 |
| Diarrhea | 0 | 0 | 1 (1.4) | 0 |
| Gastrointestinal perforation | 0 | 0 | 1 (1.4) | 0 |
| Acute hepatic failure | 0 | 0 | 1 (1.4) | 1 (1.4) |
| Hepatic function abnormal | 0 | 0 | 1 (1.4) | 1 (1.4) |
| Pneumonia aspiration | 0 | 0 | 1 (1.4) | 1 (1.4) |
| Peroneal nerve palsy | 0 | 0 | 1 (1.4) | 0 |
| Diabetes ketoacidosis | 0 | 0 | 0 | 0 |
| Pyrexia | 0 | 0 | 0 | 0 |
| Hepatitis acute | 0 | 0 | 0 | 0 |
| Bronchitis | 0 | 0 | 0 | 0 |
| Rash pustular | 0 | 0 | 0 | 0 |
| Aspartate aminotransferase increased | 0 | 0 | 0 | 0 |
| Blood bilirubin increased | 0 | 0 | 0 | 0 |
| Hepatic enzyme increased | 0 | 0 | 0 | 0 |
| Dyspnea exertional | 0 | 0 | 0 | 0 |
| Abdominal pain | 0 | 0 | 0 | 0 |
| Asthenia | 0 | 0 | 0 | 0 |
| Dermatitis | 0 | 0 | 0 | 0 |
| Sudden death | 0 | 0 | 0 | 0 |

**Supplementary Table 5** Baseline patient characteristics of Japanese patients with and without prior ramucirumab treatment.

|  | **With prior ramucirumab treatment** | | | **Without prior ramucirumab treatment** | |
| --- | --- | --- | --- | --- | --- |
|  | **Nivolumab**  **(*n* = 34)** | | **Placebo**  **(*n* = 21)** | **Nivolumab**  **(*n* = 118)** | **Placebo**  **(*n* = 53)** |
| Male | 25 (73.5) | | 15 (71.4) | 86 (72.9) | 42 (79.2) |
| Female | 9 (26.5) | | 6 (28.6) | 32 (27.1) | 11 (20.8) |
| Age (years) median (min, max) | 63.0 (45, 83) | | 65.0 (28, 79) | 66 (20, 83) | 67 (37, 78) |
| Patients aged <65 years | 18 (52.9) | | 9 (42.9) | 50 (42.4) | 19 (35.8) |
| Eastern Cooperative Oncology Group performance status | | | |  |  |
| 0 | 10 (29.4) | | 4 (19.0) | 54 (45.8) | 27 (50.9) |
| 1 | 24 (70.6) | | 17 (81.0) | 64 (54.2) | 26 (49.1) |
| Organs with metastases | | | |  |  |
| <2 | 8 (23.5) | | 7 (33.3) | 35 (29.7) | 15 (28.3) |
| ≥2 | 26 (76.5) | | 14 (66.7) | 83 (70.3) | 38 (71.7) |
| Site of metastases | |  | |  |  |
| Lymph node | 28 (82.4) | | 16 (76.2) | 101 (85.6) | 44 (83.0) |
| Peritoneum | 6 (17.6) | | 6 (28.6) | 22 (18.6) | 9 (17.0) |
| Liver | 7 (20.6) | | 3 (14.3) | 28 (23.7) | 14 (26.4) |
| Lung | 2 (5.9) | | 1 (4.8) | 9 (7.6) | 3 (5.7) |
| Pleural tissue | 0 | | 0 | 1 (0.8) | 1 (1.9) |
| Bone | 1 (2.9) | | 0 | 2 (1.7) | 3 (5.7) |
| Other | 1 (2.9) | | 3 (14.3) | 7 (5.9) | 4 (7.5) |
| Previous treatment regimens^a^ | | | |  |  |
| 2 | 2 (5.9) | | 1 (4.8) | 9 (7.6) | 2 (3.8) |
| 3 | 7 (20.6) | | 3 (14.3) | 50 (42.4) | 23 (43.4) |
| ≥4 | 25 (73.5) | | 17 (81.0) | 59 (50.0) | 28 (52.8) |
| Previous gastrectomy | | | |  |  |
| No | 11 (32.4) | | 6 (28.6) | 45 (38.1) | 25 (47.2) |
| Yes | 23 (67.6) | | 15 (71.4) | 73 (61.9) | 28 (52.8) |

Data shown are *n* (%) unless otherwise stated.

^a^Includes treatments received in the adjuvant setting.
